# Severity of Disease Among Adults Hospitalized with Laboratory-Confirmed COVID-19 Before and During the Period of SARS-CoV-2 B.1.617.2 (Delta) Predominance — COVID-NET, 14 States, January–August 2021

**DOI:** 10.15585/mmwr.mm7043e1

**Published:** 2021-10-29

**Authors:** Christopher A. Taylor, Kadam Patel, Huong Pham, Michael Whitaker, Onika Anglin, Anita K. Kambhampati, Jennifer Milucky, Shua J. Chai, Pam Daily Kirley, Nisha B. Alden, Isaac Armistead, James Meek, Kimberly Yousey-Hindes, Evan J. Anderson, Kyle P. Openo, Kenzie Teno, Andy Weigel, Maya L. Monroe, Patricia A. Ryan, Justin Henderson, Val Tellez Nunez, Erica Bye, Ruth Lynfield, Mayvilynne Poblete, Chad Smelser, Grant R. Barney, Nancy L. Spina, Nancy M. Bennett, Kevin Popham, Laurie M. Billing, Eli Shiltz, Nasreen Abdullah, Melissa Sutton, William Schaffner, H. Keipp Talbot, Jake Ortega, Andrea Price, Shikha Garg, Fiona P. Havers, COVID-NET Surveillance Team, COVID-NET Surveillance Team, Jeremy Roland, David Blythe, Alicia Brooks, Kathryn Como-Sabetti, Richard Danila, Melissa Judson, Wickliffe Omondi, Kerianne Engesser, Adam Rowe, Maria Gaitán, Virginia Cafferky, Julie Freshwater, Ann Salvator, Sam Hawkins, Emily Youngers, Tiffanie Markus, Melanie Crossland, Keegan McCaffrey

**Affiliations:** ^1^CDC COVID-19 Response Team; ^2^General Dynamics Information Technology, Atlanta, Georgia; ^3^California Emerging Infections Program, Oakland, California; ^4^Career Epidemiology Field Officer Program, CDC; ^5^Colorado Department of Public Health and Environment; ^6^Connecticut Emerging Infections Program, Yale School of Public Health, New Haven, Connecticut; ^7^Emory University School of Medicine, Atlanta, Georgia; ^8^Georgia Emerging Infections Program, Georgia Department of Health; ^9^Atlanta Veterans Affairs Medical Center, Atlanta, Georgia; ^10^Iowa Department of Public Health; ^11^Maryland Department of Health; ^12^Michigan Department of Health and Human Services; ^13^Minnesota Department of Health; ^14^New Mexico Emerging Infections Program, University of New Mexico, Albuquerque, New Mexico; ^15^New Mexico Department of Health; ^16^New York State Department of Health; ^17^University of Rochester School of Medicine and Dentistry, Rochester, New York; ^18^Rochester Emerging Infections Program, University of Rochester Medical Center, Rochester, New York; ^19^Ohio Department of Health; ^20^Public Health Division, Oregon Health Authority; ^21^Vanderbilt University Medical Center, Nashville, Tennessee; ^22^Salt Lake County Health Department.; California Emerging Infections Program, Oakland, California; Maryland Department of Health; Maryland Department of Health; Minnesota Department of Health; Minnesota Department of Health; New Mexico Department of Health; New Mexico Emerging Infections Program; New York State Department of Health; New York State Department of Health; University of Rochester School of Medicine and Dentistry, Rochester, New York; University of Rochester School of Medicine and Dentistry, Rochester, New York; Ohio Department of Health; Ohio Department of Health; Public Health Division, Oregon Health Authority; Public Health Division, Oregon Health Authority; Vanderbilt University Medical Center, Nashville, Tennessee; Salt Lake County Health Department, Salt Lake City, Utah; Utah Department of Health.

In mid-June 2021, B.1.671.2 (Delta) became the predominant variant of SARS-CoV-2, the virus that causes COVID-19, circulating in the United States. As of July 2021, the Delta variant was responsible for nearly all new SARS-CoV-2 infections in the United States.* The Delta variant is more transmissible than previously circulating SARS-CoV-2 variants (*1*); however, whether it causes more severe disease in adults has been uncertain. Data from the CDC COVID-19–Associated Hospitalization Surveillance Network (COVID-NET), a population-based surveillance system for COVID-19–associated hospitalizations, were used to examine trends in severe outcomes in adults aged ≥18 years hospitalized with laboratory-confirmed COVID-19 during periods before (January–June 2021) and during (July–August 2021) Delta variant predominance. COVID-19–associated hospitalization rates among all adults declined during January–June 2021 (pre-Delta period), before increasing during July–August 2021 (Delta period). Among sampled nonpregnant hospitalized COVID-19 patients with completed medical record abstraction and a discharge disposition during the pre-Delta period, the proportion of patients who were admitted to an intensive care unit (ICU), received invasive mechanical ventilation (IMV), or died while hospitalized did not significantly change from the pre-Delta period to the Delta period. The proportion of hospitalized COVID-19 patients who were aged 18–49 years significantly increased, from 24.7% (95% confidence interval [CI] = 23.2%–26.3%) of all hospitalizations in the pre-Delta period, to 35.8% (95% CI = 32.1%–39.5%, p<0.01) during the Delta period. When examined by vaccination status, 71.8% of COVID-19–associated hospitalizations in the Delta period were in unvaccinated adults. Adults aged 18–49 years accounted for 43.6% (95% CI = 39.1%–48.2%) of all hospitalizations among unvaccinated adults during the Delta period. No difference was observed in ICU admission, receipt of IMV, or in-hospital death among nonpregnant hospitalized adults between the pre-Delta and Delta periods. However, the proportion of unvaccinated adults aged 18–49 years hospitalized with COVID-19 has increased as the Delta variant has become more predominant. Lower vaccination coverage in this age group likely contributed to the increase in hospitalized patients during the Delta period. COVID-19 vaccination is critical for all eligible adults, including those aged <50 years who have relatively low vaccination rates compared with older adults.

COVID-NET conducts population-based surveillance for laboratory-confirmed COVID-19–associated hospitalizations in 99 counties across 14 states.^†^ Among residents of a predefined surveillance catchment area, COVID-19–associated hospitalizations are defined as a positive real-time reverse transcription–polymerase chain reaction or rapid antigen detection test result for SARS-CoV-2 during hospitalization or within the 14 days preceding admission.^§^ Unadjusted age-specific monthly population-based hospitalization rates (hospitalizations per 100,000 persons) among all adults aged ≥18 years irrespective of pregnancy status during January–August 2021 were calculated by dividing the total number of hospitalized COVID-19 patients by population estimates within each age group in the surveillance catchment area.^¶^ Using previously described methods (*2*), clinical outcomes data were collected on a representative sample of hospitalized adults stratified by age and site of admission during January–August 2021. Using a standardized case report form, trained surveillance staff members abstracted data on sampled cases (updated monthly) from medical charts that included a discharge disposition. Pregnant women (496) were excluded from the analysis because reasons for hospital admission (*3*) and standards for ICU admission might differ from those for nonpregnant persons. Severe outcomes assessed included ICU admission, receipt of IMV, and all cause in-hospital death. Severe outcomes were compared during periods before (pre-Delta period) and during Delta variant predominance (Delta period). Because COVID-19 vaccination might affect clinical outcomes (*4*), and vaccination coverage changed during the study period, results were analyzed overall and stratified by COVID-19 vaccination status.** Vaccination status was determined using state immunization information systems data (*5*,*6*). Variances were estimated using Taylor series linearization method. Chi-square testing was used to compare differences between the pre-Delta and Delta periods; p-values <0.05 were considered statistically significant, adjusted for multiple comparisons using the Bonferroni correction method. Unless otherwise noted, percentages presented are weighted to account for the probability of selection for sampled cases (*2*). All analyses were conducted using SAS statistical software survey procedures (version 9.4; SAS Institute). This activity was reviewed by CDC and was conducted consistent with applicable federal law and CDC policy.^††^

Based on 87,879 COVID-19 hospitalizations among all adults during January 1–August 31, 2021, irrespective of pregnancy status, monthly population-based rates of COVID-19–associated hospitalizations declined among all adult age groups during the pre-Delta period (Figure 1). Rates subsequently increased during July–August, with the highest rates among adults aged ≥65 years and the lowest among those aged 18–49 years. Monthly ICU admission, IMV, and in-hospital death rates followed the same patterns as COVID-19–associated hospitalization rates by age group, with the highest rates in adults aged ≥65 years and the lowest in persons aged 18–49 years.

**Figure 1:**
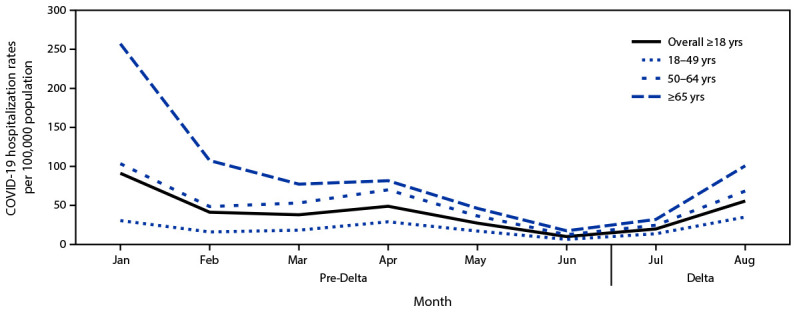
COVID-19–associated monthly hospitalization rates per 100,000 population among adults aged ≥18 years,* by age group, month, and period relative to SARS-CoV-2 B.1.617.2 (Delta) variant predominance^†^ — COVID-NET, 14 states,^§^ January–August 2021 * Proportions are from a weighted sample of hospitalized adults with completed medical chart abstraction and a discharge disposition. Results are subject to change as additional data are reported. ^†^ January–June 2021 is the pre-Delta period; the Delta period (July–August 2021) is when the Delta variant was the predominant circulating variant. ^§^ Selected counties in California, Colorado, Connecticut, Georgia, Iowa, Maryland, Michigan, Minnesota, New Mexico, New York, Ohio, Oregon, Tennessee, and Utah can be found at https://www.cdc.gov/mmwr/volumes/69/wr/mm6915e3.htm.

During January–August 2021, in a representative sample of 7,615 COVID-19 hospitalizations among nonpregnant adults with detailed clinical data available, 71.8% (weighted) of patients hospitalized during the Delta period were unvaccinated. Among unvaccinated hospitalized COVID-19 patients, the average monthly proportion who were aged 18–49 years significantly increased from 26.9% in the pre-Delta period to 43.6% during the Delta period (p<0.01) (Table). Among hospitalized COVID-19 patients who were fully vaccinated, the proportion of younger adults did not significantly change between the pre-Delta (10.6%) and Delta (10.8%) periods. Among sampled nonpregnant adults hospitalized with COVID-19, no statistically significant differences were observed between the pre-Delta and Delta periods by sex, race/ethnicity, or the proportion of patients who were admitted to an ICU, who received IMV, or who died while hospitalized, overall and stratified by age and vaccination status.

**TABLE:** Demographic characteristics and clinical interventions and outcomes among 7,615 nonpregnant adults aged ≥18 years hospitalized with COVID-19,* by vaccination status^†^ and period relative to SARS-CoV-2 B.1.617.2 (Delta) variant predominance^§^ — COVID-NET, 14 states,**^¶^** January–August 2021

Characteristic	Weighted % of COVID-19 hospitalizations (95% CI)								
	Total hospitalizations**			Unvaccinated			Fully vaccinated		
	Pre-Delta period	Delta period	p-value^††^	Pre-Delta period	Delta period	p-value^††^	Pre-Delta period	Delta period	p-value^††^
**Total**	**5,951**	**1,664**	**—**	**4,896**	**1,145**	**—**	**389**	**393**	**—**
**Demographic characteristics^§§^**									
**Age group, yrs**									
18–49	24.7 (23.2–26.3)	35.8 (32.1–39.5)	<0.01	26.9 (25.2–28.7)	43.6 (39.1–48.2)	<0.01	10.6 (6.8–15.4)	10.8 (7.1–15.4)	>0.99
50–64	31.2 (29.5–33.0)	30.4 (27.3–33.7)		32.4 (30.5–34.4)	33.6 (29.8–37.6)		17.2 (12.9–22.3)	18.8 (13.6–25.0)	
≥65	44.1 (42.0–46.2)	33.8 (30.4–37.4)		40.6 (38.3–43.0)	22.8 (19.1–26.8)		72.2 (65.8–78.0)	70.4 (63.6–76.7)	
**Sex**									
Male	52.2 (50.2–54.3)	52.3 (48.6–55.9)	>0.99	52.4 (50.2–54.6)	50.5 (46.1–55.0)	>0.99	51.7 (43.8–59.5)	56.7 (49.3–64.0)	>0.99
Female	47.8 (45.7–49.8)	47.7 (44.1–51.4)		47.6 (45.4–49.8)	49.5 (45.0–53.9)		48.3 (40.5–56.2)	43.3 (36.0–50.7)	
**Race/Ethnicity** ^¶¶^									
White	50.0 (47.9–52.0)	47.8 (44.1–51.6)	>0.99	48.5 (46.2–50.8)	45.4 (40.9–49.9)	>0.99	65.7 (57.7–73.1)	57.2 (49.2–65.0)	>0.99
Black	28.5 (26.6–30.5)	32.1 (28.5–35.9)		29.6 (27.5–31.8)	34.0 (29.6–38.7)		16.6 (10.4–24.6)	23.5 (16.8–31.3)	
AI/AN	1.1 (0.8–1.4)	1.3 (0.8–2.0)		1.0 (0.7–1.3)	1.2 (0.6–2.1)		1.2 (0.4–2.9)	1.7 (0.6–3.7)	
A/PI	6.8 (5.6–8.2)	5.4 (3.4–8.1)		7.1 (5.7–8.6)	5.0 (2.7–8.3)		5.5 (2.5–10.2)	6.8 (2.8–13.5)	
Hispanic	13.6 (12.3–15.0)	13.4 (10.9–16.2)		13.8 (12.4–15.4)	14.4 (11.3–18.0)		11.0 (7.3–15.7)	10.8 (6.4–16.8)	
Long-term care facility resident***									
Yes	7.8 (6.5–9.1)	3.2 (2.1–4.5)	<0.01	5.9 (4.7–7.3)	1.6 (0.7–3.0)^†††^	<0.01	16.7 (11.4–23.2)	8.3 (4.9–12.9)	0.59
No	92.2 (90.9–93.5)	96.8 (95.5–97.9)		94.1 (92.7–95.3)	98.4 (97.0–99.3)		83.3 (76.8–88.6)	91.7 (87.1–95.1)	
**Hospitalization interventions and outcomes, by age group, yrs^§§§^**									
**ICU admission** ^¶¶¶^									
≥18	20.1 (18.5–21.9)	23.4 (20.4–26.6)	>0.99	20.1 (18.3–21.9)	22.6 (19.1–26.3)	>0.99	19.9 (14.2–26.6)	24.6 (18.2–32.0)	>0.99
18–49	17.1 (14.6–19.9)	17.1 (12.7–22.3)	>0.99	16.8 (14.2–19.6)	16.5 (11.7–22.2)	>0.99	—****	32.0 (16.5–51.1)^††††^	>0.99
50–64	21.4 (18.9–24.1)	27.8 (22.6–33.5)	>0.99	21.4 (18.7–24.4)	27.8 (22.2–34.0)	>0.99	18.4 (10.2–29.4)	—****	>0.99
≥65	21.0 (18.1–24.1)	26.2 (20.7–32.3)	>0.99	21.1 (17.8–24.8)	26.7 (18.8–35.9)	>0.99	19.6 (12.7–28.2)	24.2 (16.5–33.4)	>0.99
**Invasive mechanical ventilation^§§§§^**									
≥18	11.5 (10.1–12.9)	11.2 (9.1–13.7)	>0.99	11.6 (10.1–13.1)	11.3 (8.8–14.2)	>0.99	9.4 (5.2–15.3)	12.7 (7.6–19.5)	>0.99
18–49	10.1 (8.1–12.4)	7.1 (4.2–10.9)	>0.99	9.7 (7.7–12.1)	7.2 (4.2–11.6)	>0.99	—****	7.4 (1.4–21.0)^†††^	>0.99
50–64	11.7 (9.8–13.9)	14.5 (10.7–19.1)	>0.99	11.7 (9.6–14.0)	16.7 (12.0–22.2)	>0.99	—****	7.0 (2.0–16.8)^†††^	>0.99
≥65	12.1 (9.7–14.8)	12.6 (8.7–17.6)	>0.99	12.7 (10.0–15.9)	11.2 (6.6–17.5)	>0.99	7.7 (3.3–15.0)	15.0 (8.3–24.3)	>0.99
**In-hospital death** ^¶¶¶¶^									
≥18	8.6 (7.5–9.9)	9.9 (7.9–12.2)	>0.99	8.2 (7.0–9.5)	8.7 (6.6–11.1)	>0.99	7.2 (4.3–11.1)	13.9 (8.7–20.7)	>0.99
18–49	3.4 (2.2–5.0)	2.0 (0.7–4.3)^†††^	>0.99	3.2 (2.0–4.9)	2.1 (0.7–4.7)^†††^	>0.99	5.0 (0.6–16.7)	2.0 (0.0–12.8)^†††^	>0.99
50–64	7.5 (5.9–9.3)	9.5 (6.4–13.5)	>0.99	7.6 (5.8–9.6)	10.5 (6.8–15.2)	>0.99	4.2 (1.0–11.3)	7.3 (1.4–20.7)^†††^	>0.99
≥65	12.3 (10.2–14.8)	18.5 (13.8–23.9)	0.70	12.0 (9.6–14.7)	18.6 (12.6–25.9)	>0.99	8.2 (4.5–13.5)	17.4 (10.5–26.3)	>0.99

**Abbreviations:** A/PI = Asian or Pacific Islander; AI/AN = American Indian or Alaska Native; CI = confidence interval; ICU = intensive care unit.

* Data are from a weighted sample of hospitalized nonpregnant adults with completed medical record abstractions and a discharge disposition. Sample sizes presented are unweighted with weighted percentages.

^†^ Vaccination status is not available for Iowa. Vaccination status is based on state immunization information system data. Fully vaccinated adults with a COVID-19–associated hospitalization were persons who had received the second dose of a 2-dose COVID-19 vaccine series or a single dose of a 1-dose product ≥14 days before receiving a positive SARS-CoV-2 test result associated with their hospitalization. Adults whose positive SARS-CoV-2 test date was ≥14 days after the first dose of a 2-dose series but <14 days after receipt of the second dose were considered partially vaccinated. Partially vaccinated adults, and those who received a single dose of a vaccine <14 days before the positive SARS-CoV-2 test result were not included in analyses by vaccination status but were included in rates and overall proportions that were not stratified by vaccination status. Adults with no documented receipt of any COVID-19 vaccine dose before the test date were considered unvaccinated. If the SARS-CoV-2 test date was not available, hospital admission date was used. Adults whose vaccination status had not yet been verified using the immunization information system data were considered to have missing vaccination status and were included in total proportions not stratified by vaccination status. Additional COVID-NET methods for determining vaccination status have been described previously. https://www.medrxiv.org/content/10.1101/2021.08.27.21262356v1

^§^ January–June 2021 is the pre-Delta period; the Delta period (July–August 2021) is when the Delta variant was the predominant circulating variant.

^¶^ Selected counties in California, Colorado, Connecticut, Georgia, Iowa, Maryland, Michigan, Minnesota, New Mexico, New York, Ohio, Oregon, Tennessee, and Utah can be found at https://www.cdc.gov/mmwr/volumes/69/wr/mm6915e3.htm.

** Total hospitalizations include data from selected counties in all 14 COVID-NET states with vaccination status, including fully vaccinated, partially vaccinated, and unvaccinated adults. As a result, the number of total hospitalizations exceeds the sum of fully vaccinated and unvaccinated adults.

^††^ Proportions between the pre-Delta and Delta period were compared with chi-square tests; p-values <0.05 were considered statistically significant, adjusted for multiple comparisons using the Bonferroni correction method.

^§§^ Percentages presented for demographic characteristics are weighted column percentages.

^¶¶^ Black, White, AI/AN, and A/PI persons were non-Hispanic; Hispanic persons could be of any race. If Hispanic ethnicity was unknown, non-Hispanic ethnicity was assumed. Persons with multiple, unknown, or missing race accounted for 3.4% (weighted) of all cases. These persons are excluded from the proportions of race/ethnicity but are otherwise included elsewhere in the analysis.

*** Long-term care facility residents include hospitalized adults who were identified as residents of a nursing home/skilled nursing facility, rehabilitation facility, assisted living/residential care, long-term acute care hospital, group/retirement home, or other long-term care facility upon hospital admission. A free-text field for other types of residences was examined; patients with a long-term care facility-type residence were also categorized as long-term care facility residents.

^†††^ Relative standard errors >30%.

^§§§^ Percentages presented for hospitalization outcomes and interventions are weighted percentages of each age group with that outcome or intervention in the pre-Delta or Delta period.

^¶¶¶^ ICU admission status was missing in 0.9% (weighted) of hospitalizations; these hospitalizations are otherwise included elsewhere in the analysis.

**** Results with relative standard errors >30% and CI widths >20 were suppressed.

^††††^ CI widths >20.

^§§§§^ Invasive of mechanical ventilation status was missing in 0.9% (weighted) of hospitalizations; these hospitalizations are otherwise included elsewhere in the analysis.

^¶¶¶¶^ In-hospital death status was missing in 1.2% (weighted) of hospitalizations; these hospitalizations are otherwise included elsewhere in the analysis.

During January–August 2021, the proportion of patients aged ≥50 years hospitalized with COVID-19 who were admitted to an ICU or who died while hospitalized generally trended upward in the Delta period (Figure 2), with the largest increase in persons who died while hospitalized among adults aged ≥65 years, (from 10.2% in June to 18.1% in August), although the difference was not statistically significant (p = 0.70). Monthly proportions of adults hospitalized with COVID-19 who received IMV also did not change significantly during January–August 2021.

**Figure 2:**
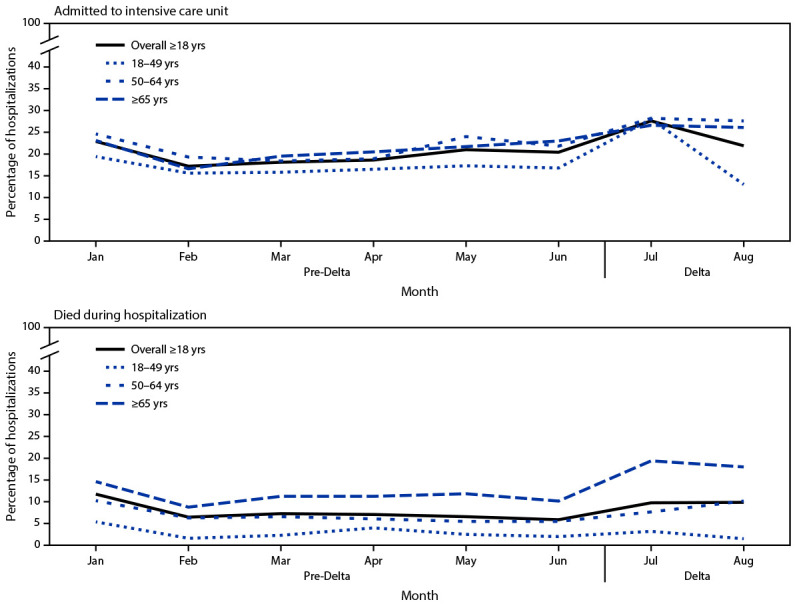
Percentage* of nonpregnant adult patients hospitalized with COVID-19 who were admitted to an intensive care unit and who died while hospitalized, by age group, month, and period relative to SARS-CoV-2 B.1.617.2 (Delta) variant predominance^†^ — COVID-NET, 14 states,^§^ January–August 2021 * Proportions are from a weighted sample of hospitalized adults with completed medical chart abstraction and a discharge disposition. Results are subject to change as additional data are reported. ^†^ January–June 2021 is the pre-Delta period; the Delta period (July–August 2021) is when the Delta variant was the predominant circulating variant. ^§^ Selected counties in California, Colorado, Connecticut, Georgia, Iowa, Maryland, Michigan, Minnesota, New Mexico, New York, Ohio, Oregon, Tennessee, and Utah can be found at https://www.cdc.gov/mmwr/volumes/69/wr/mm6915e3.htm.

## Discussion

COVID-19–associated hospitalization rates increased after the SARS-CoV-2 Delta variant became predominant. However, the proportion of nonpregnant adults aged ≥18 years hospitalized with COVID-19 who were admitted to an ICU, received IMV, or died during their hospitalization did not significantly change during this period. No significant differences in severity were observed between the pre-Delta and Delta periods among fully vaccinated or unvaccinated hospitalized patients, overall or when stratified by age and vaccination status. However, during the Delta period, adults aged 18–49 years accounted for a larger proportion of hospitalized patients compared with the pre-Delta period. This was driven by the larger number of unvaccinated hospitalized patients in this age group, likely reflecting lower vaccination coverage in younger adults than in older adults.

Similar to this analysis, a previous study examining similar outcomes during March–December 2020 (before Delta variant predominance), found that rates of ICU admission, IMV, and in-hospital death mirrored adult hospitalization rates for that period (*6*). These findings are similar to previous analyses of children and adolescents, which showed no significant differences in severe in-hospital outcomes between the pre-Delta and Delta periods (*7*,*8*). As rates of infection increased with the Delta variant, other studies have also shown increased risks for associated hospitalization (*9*,*10*), and a large Canadian study found an increased risk for ICU admission and death among a cohort of persons infected with the Delta variant (*10*). However, unlike this analysis, these studies were not limited to persons already hospitalized. Although the increasing trend in hospitalizations resulting in ICU admission or in-hospital death among adults aged ≥50 years was not statistically significant, trends in these outcomes will continue to be examined as outcomes from additional cases in later months of Delta predominance are identified.

Among unvaccinated hospitalized patients, the proportion of adults aged 18–49 years increased during the Delta period while the proportion aged ≥65 years decreased, whereas the age distribution among fully vaccinated hospitalized patients remained stable throughout the study period. All age groups included in this study were eligible to receive COVID-19 vaccines; however, as of August 31, 2021, the proportion of adults aged ≥65 years who are fully vaccinated (81.7%) is far higher than that of adults aged 18–64 years (58.6%).^§§^ Differences in vaccination coverage between age groups possibly contributed to the shift in proportional age distribution of hospitalized patients during the period of Delta predominance.

The findings in this report are subject to at least six limitations. First, COVID-19–associated hospitalizations might be undercounted because testing practices might have resulted in some persons who were admitted but did not receive testing for SARS-CoV-2. Second, the number of hospitalizations among adults aged 18–49 years is relatively small, and ICU admission, receipt of IMV, and in-hospital death are relatively rare outcomes among younger age groups, limiting the ability to examine statistical significance for some outcomes among this age group. Third, the COVID-NET surveillance catchment area represents about 10% of the U.S. population; thus, these findings should not be generalized nationally. Fourth, during periods of increased hospitalization and limited hospital capacity, clinical thresholds for hospitalization and ICU admission might shift and could potentially obscure trends in increased severity. Fifth, the analysis did not account for the propensity of persons to be vaccinated, and therefore could not determine the effectiveness of vaccination in reducing severe outcomes. Finally, data presented are preliminary and might change as additional cases are identified and reported, including cases from July and August that do not yet have a discharge disposition.^¶¶^

Rates of COVID-19–associated hospitalizations in adults increased during July–August 2021 as the Delta variant became predominant in the United States. Although this variant is more transmissible, this study did not find significantly higher proportions of hospitalizations with ICU admission, receipt of IMV, or in-hospital death in nonpregnant hospitalized adults. The proportion of unvaccinated adults aged 18–49 years hospitalized with COVID-19 has increased as the Delta variant has become more predominant. COVID-19 vaccination is critical for all eligible adults, including those aged <50 years who have relatively low vaccination rates compared with older adults.

**Summary**

**What is already known about this topic?**

The SARS-CoV-2 B.1.617.2 (Delta) variant is highly transmissible; however, whether it causes more severe disease in adults has been uncertain.

**What is added by this report?**

Analysis of COVID-NET data from 14 states found no significant increases in the proportion of hospitalized COVID-19 patients with severe outcomes during the Delta period. The proportion of hospitalized unvaccinated COVID-19 patients aged 18–49 years significantly increased during the Delta period.

**What are the implications for public health practice?**

Lower vaccination coverage in adults aged 18–49 years likely contributed to the increase in hospitalized patients during the Delta period. COVID-19 vaccination is critical for all eligible adults, including adults aged <50 years who have relatively low vaccination rates compared with older adults.

## Footnotes

*
https://covid.cdc.gov/covid-data-tracker/#variant-proportions

† Selected counties in California, Colorado, Connecticut, Georgia, Iowa, Maryland, Michigan, Minnesota, New Mexico, New York, Ohio, Oregon, Tennessee, and Utah can be found at https://www.cdc.gov/mmwr/volumes/69/wr/mm6915e3.htm.

§
https://www.cdc.gov/coronavirus/2019-ncov/covid-data/covid-net/purpose-methods.html

¶ Rates cannot be stratified by pregnancy status because the underlying population of pregnant women in the catchment area is unknown. Rates are calculated using the National Center for Health Statistics’ vintage 2019 bridged-race postcensal population estimates for the counties included in surveillance. https://www.cdc.gov/nchs/nvss/bridged_race.htm

** Fully vaccinated adults with a COVID-19–associated hospitalization were persons who had received the second dose of a 2-dose COVID-19 vaccine series or a single dose of a 1-dose product ≥14 days before receiving a positive SARS-CoV-2 test result associated with their hospitalization. Adults whose positive SARS-CoV-2 test date was ≥14 days after the first dose of a 2-dose series but <14 days after receipt of the second dose were considered partially vaccinated. Partially vaccinated adults, and those who received a single dose of a vaccine <14 days before the positive SARS-CoV-2 test result were not included in analyses by vaccination status but were included in rates and overall proportions that were not stratified by vaccination status. Adults with no documented receipt of any COVID-19 vaccine dose before the test date were considered unvaccinated. If the SARS-CoV-2 test date was not available, hospital admission date was used. Adults whose vaccination status had not yet been verified using the immunization information system data were considered to have missing vaccination status and were included in total proportions not stratified by vaccination status.

†† 45 C.F.R. part 46, 21 C.F.R. part 56; 42 U.S.C. Sect. 241(d); 5 U.S.C. Sect. 552a; 44 U.S.C. Sect. 3501 et seq.

§§
https://covid.cdc.gov/covid-data-tracker/#vaccination-demographic

¶¶ For hospitalizations with admission dates during July–August 2021, 1.3% of cases are missing a discharge diagnosis.
